# Metagenomic Sequencing of Multiple Soil Horizons and Sites in Close Vicinity Revealed Novel Secondary Metabolite Diversity

**DOI:** 10.1128/mSystems.01018-21

**Published:** 2021-10-12

**Authors:** Shrikant S. Mantri, Timo Negri, Helena Sales-Ortells, Angel Angelov, Silke Peter, Harald Neidhardt, Yvonne Oelmann, Nadine Ziemert

**Affiliations:** a Interfaculty Institute of Microbiology and Infection Medicine, University of Tübingen, Tübingen, Germany; b Interfaculty Institute for Biomedical Informatics (IBMI), University of Tübingen, Tübingen, Germany; c German Centre for Infection Research (DZIF), Partner Site Tubingen, Tübingen, Germany; d Computational Biology Laboratory, National Agri-Food Biotechnology Institute (NABI), Mohali, Punjab, India; e NGS Competence Center Tübingen (NCCT), Institut für Medizinische Mikrobiologie und Hygiene, Universitätsklinikum Tübingen, Tübingen, Germany; f Geoecology, Department of Geosciences, University of Tübingen, Tübingen, Germany; Agricultural Biotechnology Research Center

**Keywords:** amplicon sequencing, metagenome, biosynthetic gene clusters, Oxford Nanopore, soil horizons, secondary metabolites, natural products

## Abstract

Discovery of novel antibiotics is crucial for combating rapidly spreading antimicrobial resistance and new infectious diseases. Most of the clinically used antibiotics are natural products—secondary metabolites produced by soil microbes that can be cultured in the lab. Rediscovery of these secondary metabolites during discovery expeditions costs both time and resources. Metagenomics approaches can overcome this challenge by capturing both culturable and unculturable hidden microbial diversity. To be effective, such an approach should address questions like the following. Which sequencing method is better at capturing the microbial diversity and biosynthesis potential? What part of the soil should be sampled? Can patterns and correlations from such big-data explorations guide future novel natural product discovery surveys? Here, we address these questions by a paired amplicon and shotgun metagenomic sequencing survey of samples from soil horizons of multiple forest sites very close to each other. Metagenome mining identified numerous novel biosynthetic gene clusters (BGCs) and enzymatic domain sequences. Hybrid assembly of both long reads and short reads improved the metagenomic assembly and resulted in better BGC annotations. A higher percentage of novel domains was recovered from shotgun metagenome data sets than from amplicon data sets. Overall, in addition to revealing the biosynthetic potential of soil microbes, our results suggest the importance of sampling not only different soils but also their horizons to capture microbial and biosynthetic diversity and highlight the merits of metagenome sequencing methods.

**IMPORTANCE** This study helped uncover the biosynthesis potential of forest soils via exploration of shotgun metagenome and amplicon sequencing methods and showed that both methods are needed to expose the full microbial diversity in soil. Based on our metagenome mining results, we suggest revising the historical strategy of sampling soils from far-flung places, as we found a significant number of novel and diverse BGCs and domains even in different soils that are very close to each other. Furthermore, sampling of different soil horizons can reveal the additional diversity that often remains hidden and is mainly caused by differences in environmental key parameters such as soil pH and nutrient content. This paired metagenomic survey identified diversity patterns and correlations, a step toward developing a rational approach for future natural product discovery surveys.

## INTRODUCTION

One of the major driving forces of the medical revolution in the twentieth century was the discovery of antibiotics, which are often derived from secondary metabolites produced by microorganisms (1, 2). These natural products can be categorized based on their biosynthesis pathways. The major biosynthetic classes are polyketides (PKS), nonribosomal peptides (NRPS), ribosomally synthesized and posttranslationally modified peptides (RiPPs), terpenes, and saccharides. In bacteria, the genes that encode these biosynthetic pathways are clustered together in the genome, popularly termed biosynthetic gene clusters (BGCs). The genes in some of these BGCs encode modular domains and enzymes that function in an assembly line-like fashion to produce complex biomolecules. Ketosynthase (KS) and adenylation (A) domains, which have been the focus of this study, are involved in the biosynthesis of the PKS and NRPS classes of secondary metabolites in bacteria. Studying the gene sequence diversity of these domains aids in predicting the chemical structures encoded by BGCs that contain such domains (3). Based on the understanding of the biosynthetic chemical logic of these natural products, novel strategies have been developed not only to chemically synthesize analogous or derivative molecules, but also to accelerate their discovery via genome and metagenome mining methods (4–6).

Many natural products have been discovered and studied, and a collection of more than 400,000 such biomolecules is freely available from publicly accessible repositories (7, 8). These biomolecules show diverse pharmacological functions, such as antibacterial, antifungal, anticancer, immunomodulatory, and antiviral activities (9). Less characterized are their ecological functions. Multiple hypotheses and theories have been proposed about the role of secondary metabolites in the lives of the microbes that produce them. Some of these bioactive molecules are deployed in the arms race against other species in a particular microbial community; others might serve as intraspecies, interspecies, or even interkingdom, signaling and communication agents or regulate developmental processes (10).

Most of the antibiotics discovered so far have been isolated from soil microbes, specifically those that could be cultured in the lab. As research groups around the world started to extensively survey random soils to identify novel antibiotics, they experienced the challenge of rediscovering previously characterized antibiotics (11, 12). The use of 16S rRNA gene-based metagenome profiling unveiled the extent of the hidden microbial diversity, as only about 1 to 2% of all the species present in a particular soil sample could be cultured in the lab (13, 14). The subsequent revolution in next-generation sequencing (NGS) technologies made it possible not only to easily sequence the isolated species genomes, but also to capture the unculturable microbial diversity using metagenome sequencing approaches (15–17). More recently, long-read sequencing technologies, namely Oxford Nanopore and PacBio sequencing, have enabled significant improvements in the assembly of shotgun metagenomes into long contigs. These are a prerequisite for the identification of the often very large biosynthetic clusters encoding secondary metabolites. One study even reported comparable results by using only MinIon nanopore sequencing for recovering multiple complete bacterial genomes from complex microbial communities within a bioreactor (18).

The metagenomic soil surveys reported so far aimed at identifying microbial community diversity and patterns and covered areas spanning from urban green spaces and grassland meadows up to continent-wide scale soil analyses (15, 16, 19–21). A few also aimed at identifying the biosynthetic domain composition of bacterial natural products, exclusively using amplicon sequencing (amplicon-seq) approaches (22–27). Those studies were able to identify diversity patterns and correlations between natural product diversity and environmental features, thus improving our understanding of ecological and evolutionary pressures that drive the distribution of natural products across different geographical scales. However, little is known about how sampling strategies can be optimized for improved discovery of diverse natural products. Those studies that addressed these issues identified distribution patterns of PKS and NRPS based on biomes, types and characteristics of the soil (composition, pH, temperature, etc.), and geographic distance (26, 28–30). However, they analyzed the soil in either similar or different ecosystems on a global scale. Moreover, while Morlon et al. (30) identified plant community composition as the main driver of natural product diversity, Charlop-Powers (26, 28, 29) showed that geographic proximity was more important. In fact, soil types and associated soil properties may vary greatly even at a local scale (i.e., decimeters) due to differences in the geological parental material, (micro)relief, or plant community. Also, soil properties may considerably vary vertically, as different soil horizons may largely differ in physicochemical properties (e.g., pH, available nutrients, redox conditions, and water content) due to pedogenetic processes (31). As a consequence of such highly diverse microenvironments, in general microbial diversity was shown to vary by soil depth, with greater depth accompanied by decreasing abundances (32–34). Therefore, we speculated that analysis of different soil samples from different ecosystems in the same geographical area could provide more insight into the fine-scale distribution of secondary metabolites and how sampling strategies can affect natural product discovery.

Here, we report results from our metagenomics study of different horizons of soil sampled from various sites within the Schönbuch Forest, a nature reserve area in Southern Germany, using both Nanopore and Illumina NGS sequencing technologies. The major objectives of this pilot project were (i) to compare the natural product domains and biosynthesis cluster diversity of different soils and their horizons; (ii) to recover longer metagenome-assembled contigs via hybrid assembly of short and long reads, facilitating discovery of biosynthesis gene clusters; (iii) to compare the amplicon sequencing and shotgun metagenome sequencing methods; and (iv) to assess correlations between microbial community diversity and physicochemical properties of different soils. Our findings indicate that natural product diversity is high in different soils, even those in close proximity to each other, and that sampling the different soil horizons also makes a difference. Mining of metagenomic reads led to the detection of many known and novel domains involved in the biosynthesis of polyketide and nonribosomal peptides. Hybrid assembly of short and long reads led to the identification of biosynthesis gene clusters that could have never been detected by short-read sequencing alone.

## RESULTS

### Amplicon-seq mining revealed major differences in bacterial diversity and biosynthetic potential in the different soils and their horizon.

**FIG 1:**
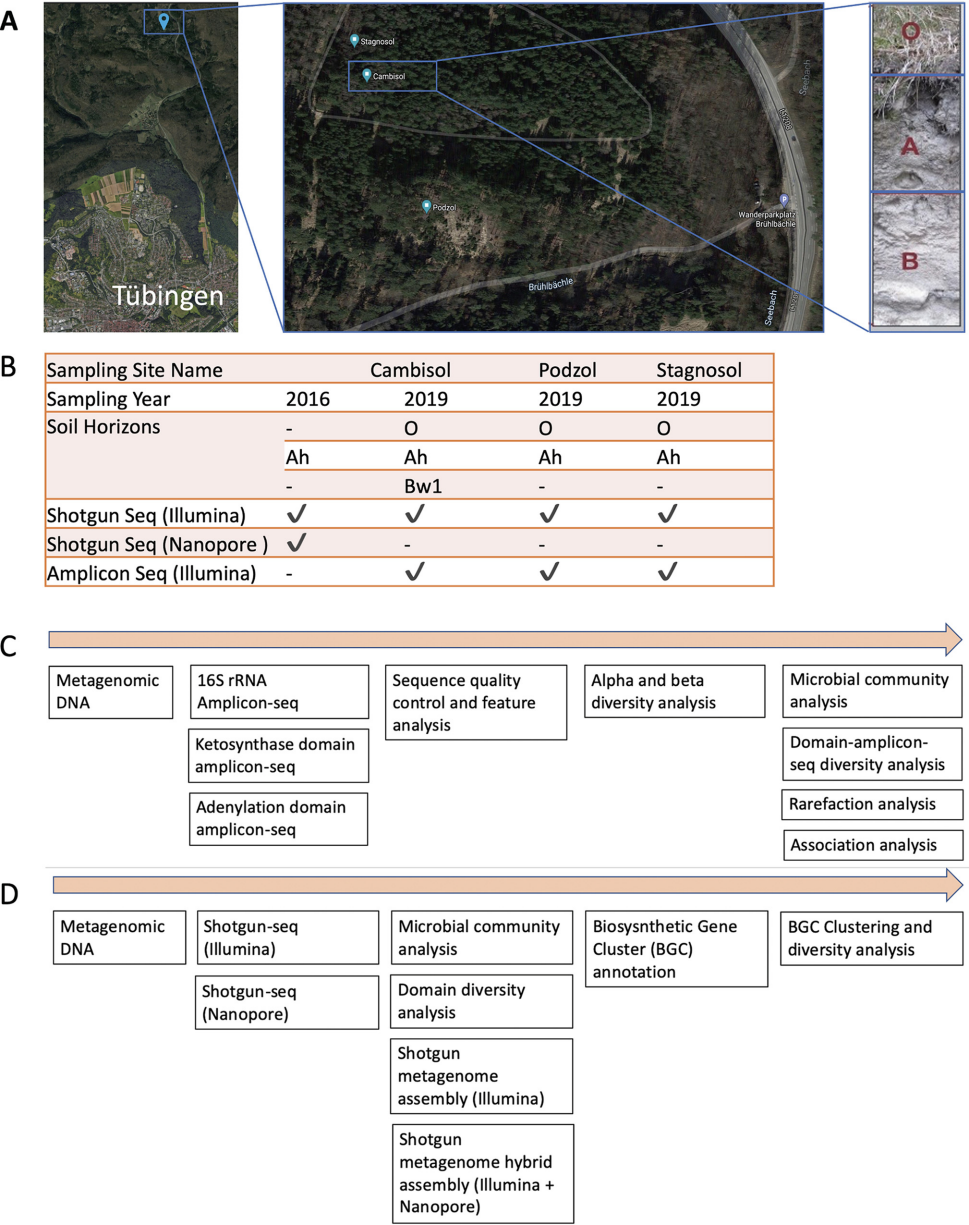
Geographic location, study outline, and analysis workflow. (A) Sampling site geographic location map of Tübingen, Germany (map data from Google ©2021). Multiple soil horizons from three sites were sampled. Photo depicting the 3 horizons of cambisol soil. (B) Sample and sequencing information, See Table S10a at https://doi.org/10.5281/zenodo.5195507 for details about soil names and profile description. (C) Amplicon sequencing (amplicon-seq) and analysis workflow. (D) Shotgun sequencing (shotgun-seq) and analysis workflow.

In order to understand how the diversity of secondary metabolites changes with the type of soil and its horizons, we identified a study area located in the Schönbuch Forest nature reserve, which is part of the South German Scarplands region (35). Soils in this area are characterized by high diversity due to a variety of geological material and landscape morphology. Samples were collected from three soil pits representing three characteristic but highly diverse soil types, namely cambisol, podzol, and stagnosol. All of the soil pits are located in a straight line within some 150 m from each other (Fig. 1A). Soil analysis has shown that these soils are heavily layered with very different parameters in each layer, and studies have shown that the bacterial diversity differs greatly, but no one knows about the secondary metabolite diversity (34). In order to get an overview of the actual domain diversity of the three different soils, all three soils and their respective horizons were sampled, and metagenomic DNA was isolated and subsequently sequenced using Illumina amplicon and shotgun sequencing (shotgun-seq) methods. Additionally, Oxford Nanopore sequencing was used to sequence one sample. Sample details, the study outline, sequencing yields, and the analysis workflow are summarized in Fig. 1 and in Table S1a to c at https://doi.org/10.5281/zenodo.5195507.

Amplicon analysis of specific genes of interest has proven to be an efficient and cost-effective strategy for metagenomic analysis. Amplifying specific genes of interest allows high coverage of these genes without extensive sequencing. Therefore, in a first approach, we explored the microbial diversity and natural products domain diversity by sequencing the 16S rRNA gene, A domain, and KS domain amplicons (biosynthetic diversity indicators) using an Illumina paired-end sequencing approach.

**FIG 2:**
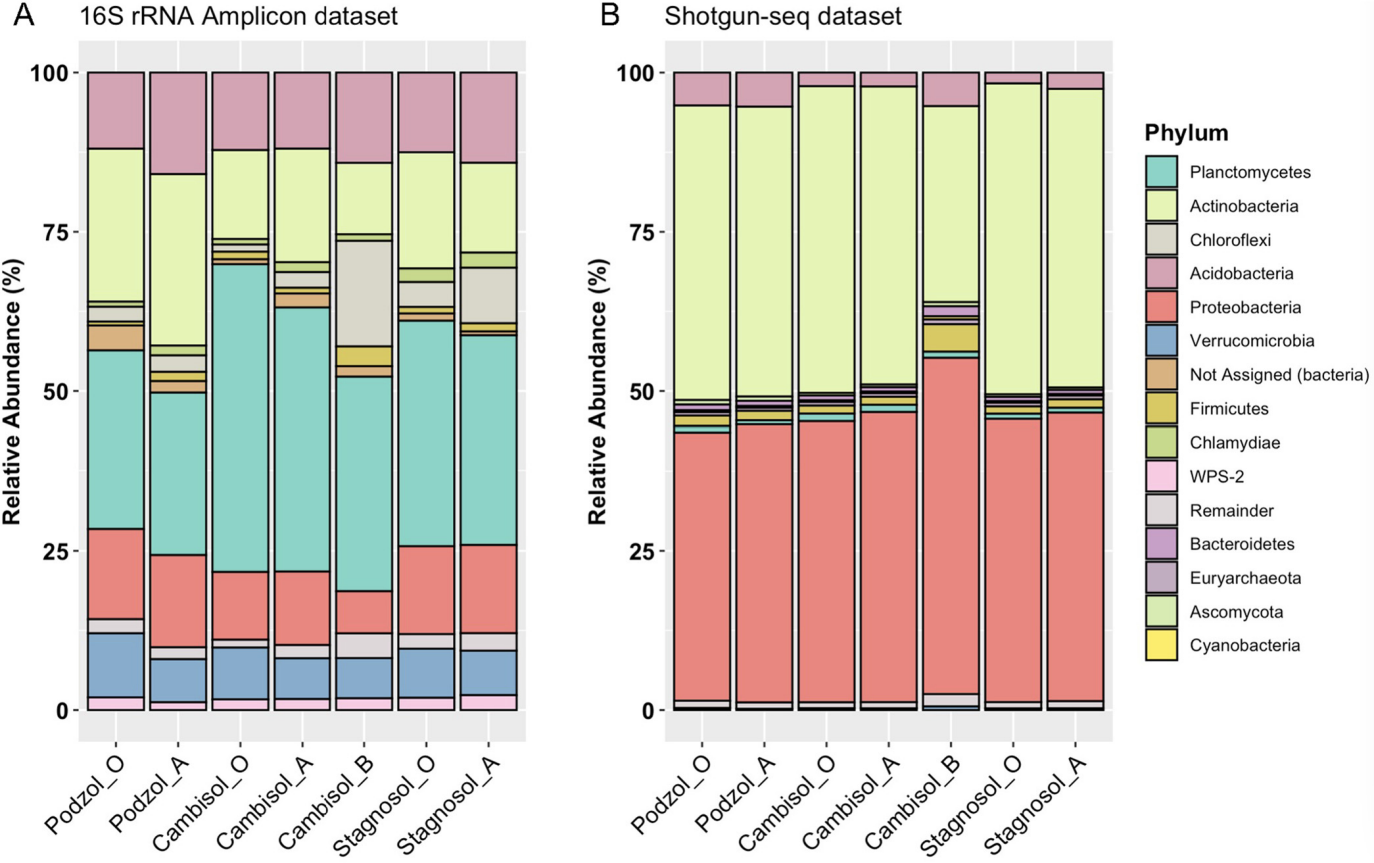
Microbial composition across 3 sampling sites (podzol, stagnosol, and cambisol) and 3 soil horizons (O, A, and B). (A) Bar plot showing taxonomic profile for 16S rRNA amplicon data set. (B) Bar plot showing taxonomic profile for shotgun-seq data set. Taxonomic profile at the phylogenetic rank of phylum is shown. The top 10 phyla are depicted in different colors, and remaining phyla are grouped as “remainder” and depicted in gray. The same colors for each phylum are used for side-by-side visualization. The SILVA rRNA database was used for classifying amplicons and the maxikraken2 database was used for classifying shotgun-seq reads.

**FIG 3:**
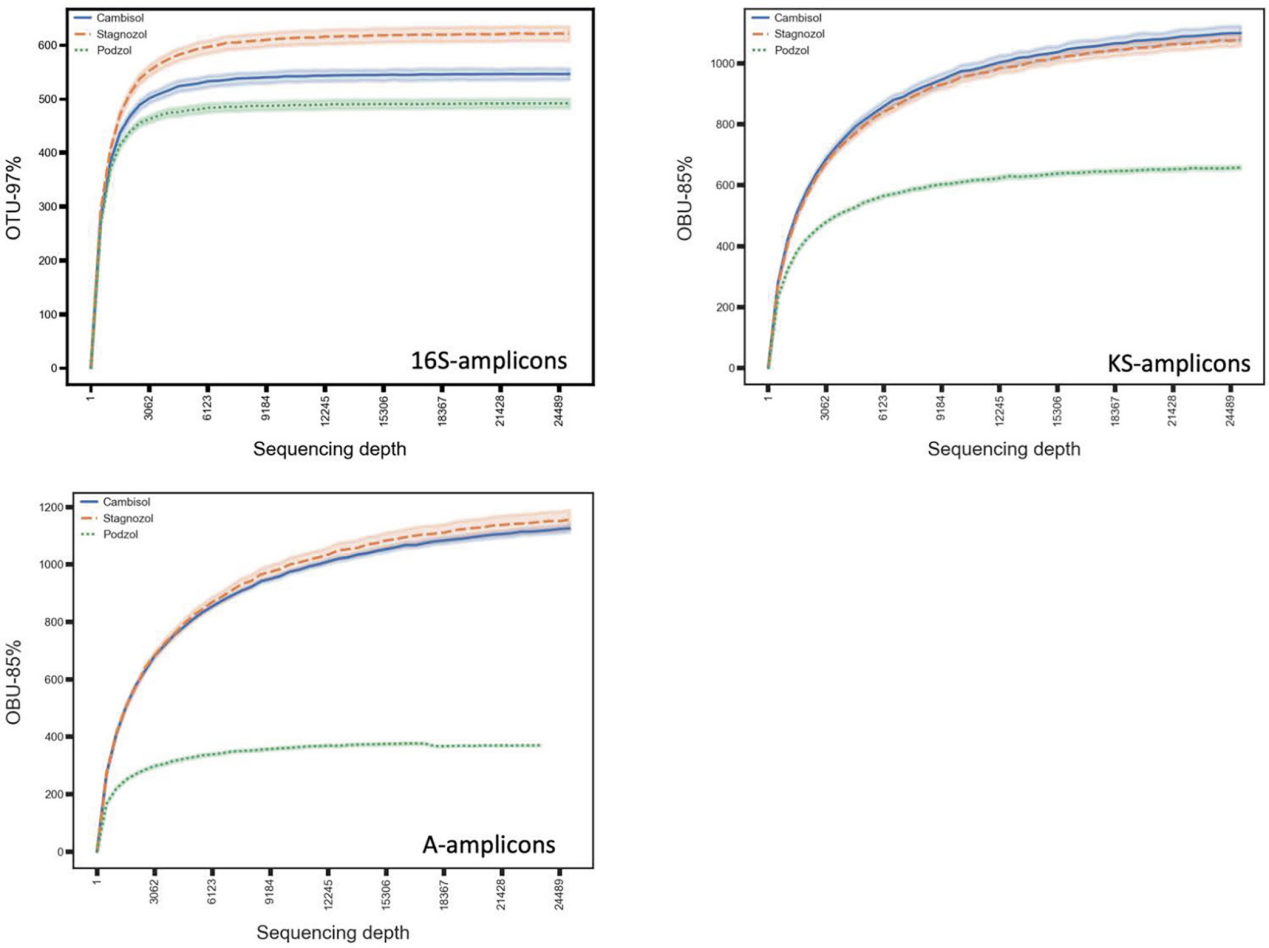
Rarefaction curves for 16S rRNA gene amplicons, adenylation (A) domain amplicons, and Ketosynthase (KS) domain amplicons. The bold curve shows mean value of operational taxonomic units (OTUs)/operational biosynthetic units (OBUs) at a particular sequencing depth for all horizons of a particular site. The faint colored area around each curve shows a confidence interval of 67%.

**FIG 4:**
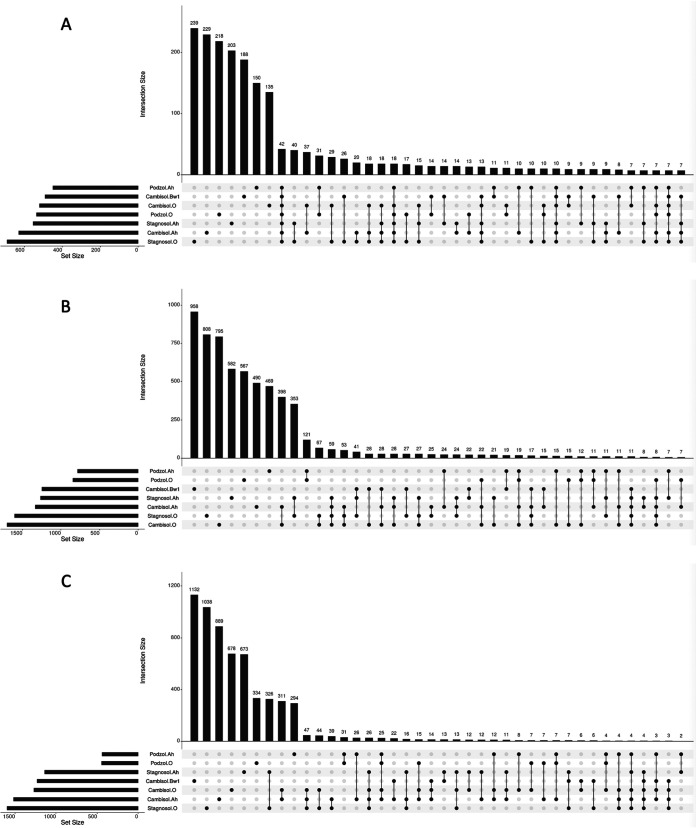
Intersections and distribution of 16S (A), KS domain (B), and A domain (C) (amplicon sequence variants [ASVs] clustered at 97% similarity). The bar plot (top) in each panel shows the intersection size (the number of ASVs) in the combinatorial sets of relevant samples. The matrix below the bar plot indicates sets of samples that are represented by each bar.

Taxonomic annotation of the Illumina-based 16S rRNA gene amplicon sequence variants (ASVs) using the SILVA taxonomic database showed that all soil samples have a very diverse bacterial composition, as expected (Fig. 2; see also Table S4a at https://doi.org/10.5281/zenodo.5195507). Comparing the taxonomic composition of all samples revealed that not only the three different soils but also their various horizons differed in their bacterial composition, even on the relatively broad phylum level (Fig. 2). Planctomycetes was the most abundant phylum in all three soil samples and all horizons. The Chloroflexi phylum was most abundant in the cambisol B horizon, with a relative frequency double that in other soils. By comparing the number of ASVs and clustering them in operational taxonomic units (OTUs), we noticed that the highest number of OTUs was present in the A horizon of cambisol, which represents the second layer below the surface (see Table S6a at https://doi.org/10.5281/zenodo.5195507). In contrast, in podzol and stagnosol, the numbers of OTUs in the O horizons were higher than those in the respective A horizons. The lowest number of OTUs was found in the cambisol B horizon, indicating that cambisol contained the most but also the least bacterial diversity of the three different soils, depending on the horizon. In order to classify A domain and KS domain amplicons into groups that represent distinct chemical classes and biosynthetic gene clusters (BGCs), we clustered these amplicons into operational biosynthetic units (OBUs) as previously described (24). Rarefaction curve analysis for both classes of OBUs showed that the curves are still ascending, indicating that the full biosynthetic diversity has not yet been captured, in contrast to the taxonomic diversity represented by the 16s rRNA amplicons (Fig. 3). Comparing the domain diversity of the different soils and their horizons showed that unique KS and A domains (ASVs clustered at 97% similarity; see Fig. 4) were at a maximum in the cambisol B horizon, the soil with the lowest number of OTUs (see Table S6a at https://doi.org/10.5281/zenodo.5195507). In order to uncover any possible correlation between biosynthetic diversity and taxonomic diversity, we compared various alpha diversity indices of KS and A domains with the 16S diversity. The OTU alpha diversity, Faith phylogenetic diversity (PD), Shannon, and evenness indices showed high correlation across 16S and A domain amplicons (see Table S6a at https://doi.org/10.5281/zenodo.5195507), whereas there was no clear correlation for KS domains, and a negative correlation between evenness of 16S and KS domains was observed.

In order to disclose any overlap between the different soils, we compared 16S as well as KS and A domain amplicons in the different samples using UpSet plots (Fig. 4). This analysis revealed that, while there was an overlap of 42 16S amplicons across all the 7 samples, no such degree of sequence similarity was observed for KS and A domains. ASVs of these domains were only conserved between samples of different horizons of the same site.

To see if the differences in taxonomic diversity and biosynthetic potential of the different soil samples were correlated with the unique soil physicochemical parameters, we calculated alpha diversity (16S and domain) correlations with the soil parameters (see Table S6b at https://doi.org/10.5281/zenodo.5195507). Although we were able to detect some correlations with biosynthetic potential—pH showed a close correlation between KS domain alpha diversity measures (Shannon *r* = 0.75, *P* = 0.05; evenness *r* = 0.78, *P* = 0.03)—we think that more data are needed in order to properly interpret these results.

### Shotgun metagenome mining further uncovered microbial diversity and identified novel BGCs.

Amplicon sequencing-based studies of metagenome diversity are an economical approach; however, the limitations of this approach became evident when we performed shotgun metagenome sequencing using Illumina short reads and Nanopore long reads for the same samples and compared both methods.

We used the Kraken 2 algorithm in order to annotate the shotgun metagenomes, which led to an average of 47.04% classified reads and an average of 52.95% unclassified reads (Table 1). Interestingly, Proteobacteria and Actinobacteria were the two most frequently annotated phyla among all the metagenomes (Fig. 2), a result which differs greatly from the 16S rRNA gene amplicon annotations. Using the unassembled metagenomes, we also used BiG-MEx software for annotations of BGC domains and diversity analysis. BiG-MEx was able to annotate 150 BGC domains (see Table S5b at https://doi.org/10.5281/zenodo.5195507), most of them as A domains. By performing comparative analysis of KS and A domains captured via amplicon and shotgun metagenome sequencing, we found that more than 90% of domains detected in shotgun metagenomes could not be detected using amplicon sequencing. More precisely, sequence similarity analysis between domains identified via amplicon sequencing and shotgun metagenome sequencing revealed the presence of domains unique to each of the methods. A total of 638 KS amplicon-seq amplicons did not show similarity to any of the KS shotgun-seq OBUs, whereas 1,571 A domain amplicon-seq amplicons did not show similarity to any of the 181,324 A domain shotgun-seq OBUs (see Table S9 at https://doi.org/10.5281/zenodo.5195507). The alpha diversity comparisons between microbial community diversity and biosynthetic domain diversity showed a diverse pattern for each domain. We also found no concurrence of these diversity correlations between amplicon-seq and shotgun-seq data sets.

**TABLE 1:** Taxonomic annotation summary^*a*^ of shotgun-seq Illumina metagenomes

Name	No. of raw paired-end reads	Classified reads (%)	Unclassified reads (%)	Microbial reads (%)	Bacterial reads (%)	Viral reads (%)
Podzol-O	113,350,452	43.90	56.10	43.80	42.90	0.01
Podzol-A	86,440,710	45.80	54.20	45.80	44.90	0.01
Cambisol-O	82,298,268	51.60	48.40	51.60	50.70	0.01
Cambisol-A	71,637,596	50.30	49.70	50.20	49.40	0.01
Cambisol-B	75,654,703	35.30	64.70	35.30	34.40	0.01
Stagnosol-O	64,281,069	52.50	47.50	52.50	51.50	0.01
Stagnosol-A	53,255,349	49.90	50.10	49.90	49	0.01

a Tool: Kraken 2; database: maxikraken2.

**FIG 5:**
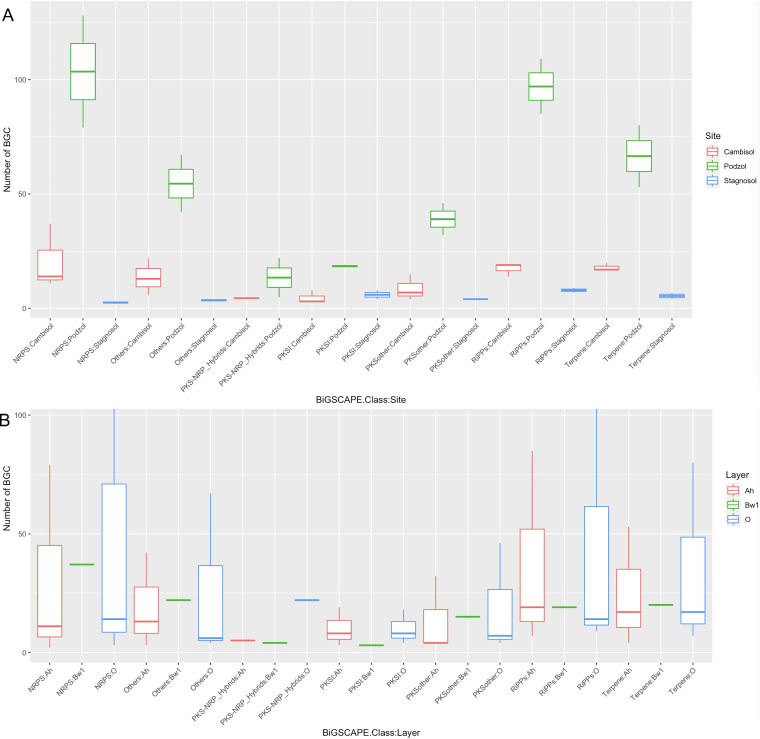
Biosynthetic gene cluster (BGC) abundance distribution. (A) BGC abundance distribution across soil sampling sites (grouped according to BiG-SCAPE class). (B) BGC abundance distribution across soil horizons.

As a next step, we assembled the shotgun metagenome data to recover full biosynthetic gene cluster sequences and thus obtain more valuable information about the encoded compounds. The metaSPAdes-based assembly of Illumina reads of all the metagenomic samples led to a total of more than 2 million contigs longer than 1 kb. The total length of all the contigs exceeded 9 Gb, with a largest contig of about 3.5 Mb. The assembled contigs longer than 10 kb were analyzed for the presence of BGCs using antiSMASH (version 5). A total of 1,102 BGCs were identified. The detailed biosynthetic class-wise breakdown of the BGC annotation is provided in Fig. 5. Again, the largest number of BGCs were annotated as belonging to the NRPS class, followed by 262 RiPPs (see Table S7a at https://doi.org/10.5281/zenodo.5195507). The podzol O horizon contained a maximum number of 470 BGCs, followed by the podzol A horizon with 315 BGCs (Fig. 5). In contrast to the domain analysis, podzol samples displayed the maximum number of clusters compared to other sites. However, this might be due to the better assembly of podzol samples as a result of the highest number of reads being generated from the O and A horizons of podzol soil (see Table S1a at https://doi.org/10.5281/zenodo.5195507). BiG-SCAPE clustering of the data set composed of only Illumina-assembled contigs helped investigate the overlap of clusters across the soil. While most of the BGCs were unique to each sample, we found only a single gene cluster family (GCF) containing BGCs from each of the seven samples. This GCF belongs to the class of terpenes.

Apart from antiSMASH-based BGC discovery, we also explored a machine learning-based method for novel cluster discovery and annotation. We found around 22,194 putative BGCs in the metagenomic contigs using the DeepBGC tool. For 7,295 of these BGCs, the biosynthesis class could be predicted. Biological activity could be predicted in 17,032 putative BGCs (see Table S8 at https://doi.org/10.5281/zenodo.5195507). While the number of detected BGCs is severalfold higher than that annotated via antiSMASH, it will be interesting to see wet-lab validation of these clusters in future studies. Although absolute numbers of predicted BGCs differed between antiSMASH and DeepBGC, the largest number of BGCs was predicted in podzol samples by both these tools.

### Comparative analysis highlights the advantage of long reads to capture biosynthetic potential.

The assembly statistics of the short-read shotgun data helped appreciate its advantages and limits. Subsequently, as we were interested in assessing how long-read Nanopore data would improve the recovery of BGCs, we performed a metaSPAdes-based hybrid assembly of Illumina and Nanopore reads of the cambisol A metagenome. The hybrid assembly substantially enhanced the overall length of the contigs and the number of longer contigs. We found seven times more hybrid contigs of lengths greater than 50 kb than Illumina-only contigs of the same length. The largest hybrid contig was 598,670 bases (see Table S3a and b at https://doi.org/10.5281/zenodo.5195507). AntiSMASH analysis resulted in the annotation of 169 BGCs among the hybrid contigs longer than 10 kb. This is more than double the number of BGCs that were found in Illumina-only contigs. A total of 1,026 BGCs with lengths greater than 1 kb were even annotated in the hybrid contigs. Comparison of metagenomic contig length (Illumina-only versus hybrid data) revealed substantial improvements with the hybrid assembly approach (see Table S3a and b at https://doi.org/10.5281/zenodo.5195507. In several instances, hybrid assembly enabled the extension of Illumina contigs containing BGCs, thus making it possible to determine whether resistance markers or regulator-encoding genes were present within the clusters. We found more than 2-fold more BGCs in hybrid contigs that were not on contig borders than Illumina-only contigs detected via antiSMASH annotation. We also performed BiG-SCAPE clustering of all BGCs from Illumina and hybrid metagenomes to identify BGCs that were detected in multiple samples. This analysis led to the identification of 1,803 GCFs. A total of 1,625 GCFs contained only single members (see Table S7b at https://doi.org/10.5281/zenodo.5195507).

## DISCUSSION

Soil formation is a slow process. Depending on climatic conditions; it might take several hundred years to form just a 1-cm layer of soil. While most of the antibiotics discovered so far have been largely isolated from culturable microbes in random sampling of topsoils, the immense metabolic diversity of unculturable microbial dark matter in both topsoils and deeper soil horizons has remained largely hidden (36). As the depth of the soil increases, the organic and inorganic chemical constituents and morphology of soil change drastically, creating microenvironments that can accelerate the evolution of novel microbial species (37). To capture the biosynthetic novelty of all such microbes, those that were born due to serendipitous events and those that survived the so-called microbial arms race, we decided to broaden the soil surveys not only to include soils from different sites but also to cover sampling of diverse soil horizons (38). Our study is also unique in that it used both amplicon sequencing and shotgun metagenome sequencing of the same soil samples to determine the biosynthetic potential that a particular site and ecosystem hold and to discover novel natural product domains and BGCs (Fig. 1).

Although a few species were ubiquitously present across all the sites and all the soil horizon layers, a significantly higher proportion of OTUs/species were seen to be unique to individual samples (Fig. 4a). BGC domain diversity and distribution observed across all the samples indicate higher overlap within a particular sampling site than across sites (Fig. 4b and c). Our survey of multiple soil horizons from multiple sites helped appreciate the presence of high vertical diversity (differences between O, A, and B horizons of each soil type), emphasizing the importance of sampling not only different geographical sites but also the vertical diversity present in different soil horizons. This is in line with previous findings based on 16S rRNA analysis (34). The reasons behind such great diversity across sites could be attributed to varied environmental conditions (33). For example, podzol is an extremely nutrient-poor, acidic, and water-scarce environment in which microbial decomposition of the tree litter is so much hampered that a thick organic litter layer sits on top of the topsoil (i.e., A horizon); in stagnosol A and B horizons, instead, the water dynamics can become entirely dry during summer, changing the redox from reducing to oxic.

Drastic deviations in estimating microbial composition via both 16S amplicon-seq and shotgun-seq have been previously reported (39, 40). In our study, Planctomycetes emerged as the major phylum in the amplicon-seq analysis, while Proteobacteria and Actinobacteria were the predominant phyla in the shotgun-seq analysis (Fig. 2). This deviation could be attributed to primer and PCR bias of the 16S amplicon method (39, 40) and to the different bioinformatics workflows (41). Also, the sequencing depth in studying the microbial composition via 16S amplicon sequencing appeared to be sufficient and saturating based on the rarefaction curves (Fig. 3). Subsequent shotgun metagenome sequencing analysis of the same samples revealed that amplicon-based analysis underestimated the alpha diversity of the samples.

Although we hoped to find unique patterns of correlations between microbial community diversity and biosynthetic diversity, our results from both amplicon-seq and shotgun-seq data sets only revealed few correlations with few biosynthetic gene domains. We speculate that these patterns would become more evident as more optimized amplicon primers, capable of amplifying additional biosynthetic genes and their domains, become available. In the case of shotgun-seq data sets, higher depth of sequencing of the samples would not only help in recovering more full-length BGCs but also in revealing biosynthesis domain diversity patterns. Better software tools capable of handling such high volumes of data would be required to mine the biosynthetic diversity patterns.

Assembly of shotgun-seq Illumina reads followed by antiSMASH annotation led to the discovery of 1,102 BGCs. *Proteobacteria*, Acidobacteria, and Actinobacteria were the major phyla to which many of these BGCs were taxonomically annotated. Distribution patterns of BGC classes across the sampling sites and soil horizons show that the podzol site has the maximum number of BGCs (Fig. 5). BGC abundance distributions were observed to be greater in sampling site-wise comparison than in soil layer-wise comparison. BGC clustering analysis also revealed how different the various samples and horizons are, as only a single BGC was found to be present across all the samples (see Fig. S1 at https://doi.org/10.5281/zenodo.5195507). Hybrid assembly of Illumina short reads with Nanopore long reads led to the recovery of complete BGCs in some cases, enabling the identification of the regulatory genes and resistance genes in the vicinity of the identified BGCs. Such proximity analysis can be helpful in prioritizing the BGCs for, e.g., the characterization of the encoded compounds in heterologous expression systems (42). Machine learning-based annotation of assembled contigs using DeepBGC led to identification of even more putative BGCs. For many of them, however, the biosynthesis class and activity could not be predicted, likely as a consequence of the low similarity between these novel BGCs and those used for DeepBGC training.

Amplicon sequencing and shotgun metagenome sequencing are both important when aiming for novel domain discovery, as we observed unique domain sequences with each of the methods (see Table S9 at https://doi.org/10.5281/zenodo.5195507). For both KS and A domains, 90% more domain sequences were identified in shotgun data sets than in amplicon data sets, highlighting the immense biosynthesis potential that has yet to be discovered. As the costs of shotgun metagenomic sequencing are still prohibitive and make these methods accessible to only a few, our shotgun results will be useful to design domain sequence-based primers that are not biased to a particular genus and can be used for massive amplicon-based diversity surveys.

Our study helped capture a snapshot of microbial diversity and metabolic novelty from the soils sampled on a single day. However, the limited number of samples made it hard to draw meaningful biological conclusions from the observed correlations between the diversity of BGCs and soil physicochemical parameters. Large-scale and more systematic sampling across changing weather or seasons will be necessary to capture the true dynamics and complete diversity. We were not able to recover metagenome-assembled genomes (MAGs) due to sequencing volume limitation. Considering the massive diversity present in soil, hundreds if not thousands of gigabases would be required to reach a stage to claim complete coverage of all of the species genomes in a particular metagenome sample (43). Reaching terabase scales (10^12^) is not only a current economical bottleneck, but also calls for better metagenome assembly algorithms that are both space and time efficient. Alternatively, a novel method that uses live fluorescence *in situ* hybridization (FISH) combined with fluorescence-activated cell sorting (FACS) has been reported to be capable of isolating live bacteria based solely on their 16S rRNA gene sequence (44). In future, using such novel methods will make it possible to accelerate the BGC discovery from candidate or novel phyla present in densely rich soil samples.

Some of the BGCs discovered in this study are currently being explored for further heterologous expression and structure elucidation in our laboratory. All of the data resources generated here have been shared in the public domain to facilitate further experiments and analysis by the natural products research community. It will be a herculean task to explore and map the complete chemical space that natural products cover on the entire earth. Our metagenomic data give a glimpse of the immense microbial and biosynthetic diversity that exists even in next-door soils.

### Conclusion.

Overall, this study helped uncover the biosynthesis potential of the Schönbuch Forest soil by combining metagenome and amplicon sequencing. This paired strategy helped identify more novel BGC domains than would have been possible with either of the sequencing methods alone. Our analysis also confirmed the limitations of amplicon sequencing, which is extremely powerful in providing a glimpse of the microbial and biosynthetic diversity in soil samples, but this is biased toward sequences that are abundant in the samples and toward the chosen primers. We show that a shotgun metagenome approach is able to overcome these limitations and is better than the amplicon-based approach at capturing the microbial diversity. The additional use of Nanopore sequencing data for one of the soil samples allowed us to improve metagenome assembly and to recover novel BGCs. Nonetheless, long-read sequencing remains too costly to be routinely used in soil surveys of microbial and BGC diversity. Physicochemical parameters that correlate with the domains or BGC diversity will help develop a rationale to guide such explorative surveys. In the future, sequencing terabases of metagenomes might become feasible and economical. At such sequencing depths we might then only be limited by heterologous expression and functional validation of novel natural products. Such a foreseeable future is probably just a decade away. Until then, the approaches and rationale developed here will help fuel the drug discovery pipeline to combat antimicrobial resistance.

## MATERIALS AND METHODS

### Soil sampling and physicochemical parameter characterization.

The sampled Schönbuch Forest soils developed from Lower and Middle Triassic Keuper sequences, which locally comprise thin sequences of sandstones and evaporitic marlstones, as well as aeolian (loess), colluvial, and alluvial deposits (35, 45). The soils were described and classified according to the classification system of the Food and Agriculture Organization of the United Nations (46) and IUSS Working Group WRB (31). Differences concerning the geochemistry (i.e., pH and CaCO_3_ concentrations) of the geological soil parent material resulted in highly different soil types, which were explicitly taken into account in this study. The first soil pit, located at the top slope of a south-exposed slope was classified as a podzol, which developed from a sandstone outcrop. The second soil was classified as a cambisol, which developed from sandstone mixed with aeolian deposits (loess). The third soil was a stagnosol, which formed from a clay-rich marl. For further details on the soil profiles, see Table S10a at https://doi.org/10.5281/zenodo.5195507. Sampling was carried out by horizon. Bulk samples were taken from the soil genetic horizons for geochemical analyses, comprising the mineral topsoil (A horizon) and mineral subsoil (B horizon). For simplification, the organic litter layers (Oi and Oe) that cover the mineral soil horizons were combined as one bulk sample per site.

### (i) Carbon and nitrogen measurements.

Dried (40°C) litter and fine soil (<2 mm) samples were homogenized with a planetary ball mill (Pulverisette 5; Fritsch, Idar-Oberstein, Germany). Total C and N concentrations were measured by a CNS elemental analyzer (Vario EL III; Elementar Analysensysteme GmbH, Langenselbold, Germany). For details regarding detection limits and quality controls, see Table S10b at https://doi.org/10.5281/zenodo.5195507.

### (ii) X-ray fluorescence.

To determine the major element concentrations in fine mineral soil samples of A and B horizons, glass beads of a homogenized mixture of 1.5 g dried and powdered sample material and 7.5 g lithium tetraborate were fused at 1,050°C for 30 min. On a Bruker AXS Pioneer S4 instrument, glass beads were analyzed by wavelength-dispersive X-ray fluorescence (XRF).

### (iii) inductively coupled plasma optical emission spectrometry.

To determine concentrations of major and trace elements in O-horizon soils, litter samples were dissolved by an acid pressure digestion system (PDS-6; Loftfield Analytical Solutions, Neu Eichenberg, Germany). Homogenized sample material (target wt, 0.05 g) was transferred into Teflon pressure beakers before adding 4 ml HNO_3_ concentrate (65%, pro analysis ≥98%; Merck KGaA). After heating for 7 h at 180°C, digestion solutions were filtered (MN 619 G, 185-mm diameter; Macherey-Nagel, Düren, Germany) and diluted with Millipore water (Synergy UV ultrapure) to a final volume of 50 ml. The digests were finally analyzed using inductively coupled plasma optical emission spectrometry (ICP-OES) (Optima 5300 DV, PerkinElmer, Wellesley Hills, MA) according to EN ISO 11885. To check for accuracy and precision of the digestions, the two certified reference materials BCR-129 (hay powder) and BCR-141 (plankton) were used. Based on the measured average concentration values and the target values, recovery rates were calculated for each element (see Table S10c at https://doi.org/10.5281/zenodo.5195507). Despite a good reproducibility (relative standard deviation of 5% to 11%), most major and trace elements in BCR-129 and 141 were systematically underestimated by up to 30% (see Table S10c at https://doi.org/10.5281/zenodo.5195507), which is why correction factors were calculated and applied to the other samples. Additional analytical information is provided in Table S10c at https://doi.org/10.5281/zenodo.5195507. All vessels used were soaked in 10% HCl overnight and rinsed with Millipore water prior to use.

### (iv) Soil sampling for Nanopore/Illumina sequencing.

The A horizon of the soil type cambisol used for high-molecular-weight (HMW) DNA isolation for subsequent sequencing was sampled from the Schönbuch Forest in November 2016, transported to the lab, and stored at −20°C.

### (v) Soil sampling for Illumina and amplicon sequencing of 7 soil samples.

The O and A horizon of the soil types podzol and stagnosol, as well as the O, A, and B horizons of cambisol soil were sampled from the Schönbuch Forest on 3 May 2019. Samples were collected using a soil probe, transported to the lab, and stored at −20°C. To obtain the fine soil fraction, all soil samples were passed through a coarse mesh screen (1.2 cm × 1.2 cm) and subsequently through a fine mesh screen (2 mm × 2 mm) prior to metagenomic DNA isolation.

### Metagenome sequencing.

### (i) Isolation of HMW DNA from the A horizon of cambisol for Nanopore sequencing run 1.

HMW DNA was isolated from thawed fine soil samples using a published protocol (48) with the following modification to increase the purity of the isolated DNA. After electroelution of the DNA out of the gel and into the dialysis bag, the dialysis bag was incubated in 0.5× Tris-EDTA (TE) buffer overnight before following the next steps of the protocol. Library preparation and Nanopore sequencing of the isolated DNA were performed by genXone, Inc., on a GridIon device.

### (ii) Isolation of HMW DNA from the A horizon of cambisol for Illumina sequencing.

For Illumina sequencing, the above-described DNA sample was further purified using the spin columns of the PowerLyzer PowerSoil DNA isolation kit (catalog no. 12855-100; Mo Bio Laboratories, Inc.) and following an alternative protocol that was provided by Mo Bio. The DNA sample isolated for Nanopore sequencing run 1 was filled up to 650 μl with H_2_O, and 650 μl of solution C4 and 650 μl of 100% ethanol were added. A 650-μl aliquot of the mixture was loaded onto a Mo Bio spin column, and DNA was bound in three steps by centrifugation. The membrane was washed with 650 μl of 100% ethanol and subsequently with 500 μl of solution C5. The spin column was dried by centrifugation for 2 min at full speed and transferred to a clean tube. DNA was eluted with H_2_O. Library preparation (TrueSeq DNA PCR-Free) and Illumina sequencing were performed by CeGaT GmbH on a NovaSeq 6000 PE150 instrument.

### (iii) Isolation of HMW DNA from the A horizon of cambisol for Nanopore sequencing run 2.

HMW DNA was isolated from 6 × 5 g of thawed fine soil using a published protocol (47) with the following modifications to increase DNA yield and purity. After dissolving the dried pellets in 1 ml of 1× TE buffer, 1 μl of RNase I was added and incubated for 30 min at 37°C before following the next steps of the protocol. In addition to precipitating the DNA with a 0.7 volume of isopropanol, a 0.1 volume of 5 M sodium acetate was added. After completing the protocol, the DNA was further gel purified as described by Brady (48) and adding a dialysis step in 0.5× TE overnight after electroelution of the DNA out of the gel and into the dialysis bag. Library preparation (native ligation sequencing kit, SQK-LSK109) and sequencing were performed by the NGS Competence Center Tübingen (NCCT) on a PromethION device.

### (iv) Isolation of metagenomic DNA from 7 soil samples for Illumina sequencing.

Metagenomic DNA was isolated from the O and A horizons of the podzol, cambisol, and stagnosol sites using the PowerLyzer PowerSoil DNA isolation kit (catalog no. 12855-100; Mo Bio Laboratories, Inc.) and following an alternative protocol that was provided by Mo Bio, in which 250 mg of each thawed fine soil sample was added to dry glass bead tubes and 500 μl of bead solution and 200 μl of phenol-chloroform/isoamyl alcohol were added, followed by 60 μl of solution C1. Cells were opened using a Precellys 24 device (6,500 rpm, 2 cycles of 20 s with 5-s pause) followed by centrifugation to the pellet. The supernatant was transferred to a new tube, and 5 μl of RNase A was added as an additional step not mentioned in the protocol. Then, 250 μl of solution C2, followed by 100 μl of solution C3, was added and mixed. The mixture was incubated for 5 min at 4°C and subsequently centrifuged to the pellet. The supernatant was transferred to a new tube, and 650 μl of solution C4 and 650 μl of 100% ethanol were added. A 650-μl aliquot of the mixture was loaded onto a Mo Bio spin column, and DNA was bound in three steps by centrifugation. The membrane was washed with 650 μl of 100% ethanol and subsequently with 500 μl of solution C5 in the case of unstained membranes. In the case of brown membranes, a mixture of 300 μl solution C4 and 370 μl 100% ethanol was used to wash the membrane before washing with 100% ethanol and solution C5. The spin column was dried by centrifugation for 2 min at full speed and transferred to a clean tube. DNA was eluted with H_2_O. Metagenomic DNA from the B horizon of the cambisol was isolated following the protocol of Verma et al. (47) with the above-mentioned modifications. Library preparation (TrueSeq DNA PCR-Free) and Illumina sequencing were performed by CeGaT GmbH on a NovaSeq 6000 PE150 instrument.

### (v) Amplicon sequencing.

Isolated metagenomic DNA of the 7 soil samples and published degenerate primers that recognize conserved regions in NRPS A domains (Adom_fw: GCSTACSYSATSTACACSTCSGG; Adom_rv: SASGTCVCCSGTSCGGTAS) (49), PKSI KSI domains (KSI_fw: CCSCAGSAGCGCSTSYTSCTSGA; KSI_rv: GTSCCSGTSCCGTGSGYSTCSA) (50) and 16S rRNA genes (16S_fw: CCTACGGGNGGCWGCAG; 16S_rv: GACTACHVGGGTATCTAATCC) (51) were used to generate amplicons via PCR. Concentrations of the DNA extracted from each of the 7 soil samples was measured using a Qubit 3.0 fluorometer and adjusted to 1.5 ng/μl. PCR was performed using the Q5 high-fidelity DNA polymerase kt (NEB) with the following reaction setup for a 25-μl reaction mixture: 5 μl of 5× Q5 reaction buffer, 0.5 μl of 10 mM deoxynucleoside triphosphates (dNTPs), 0.5 μl of 10 μM forward/reverse primer, 3 μl of template DNA, 0.25 μl of Q5 high-fidelity DNA polymerase, 5 μl of 5× Q5 High GC Enhancer, and 10.25 μl of nuclease-free water. The following thermocycling conditions were used: 98°C for 30 s followed by 30 cycles of 98°C for 10 s, 58.5°C (A domain) or 68°C (KSI domain, 16S rRNA gene) for 30 s, 72°C for 20 s, and a final step with 72°C for 2 min. For each soil and primer pair, four 25-μl reactions were performed. A 5-μl aliquot of each was analyzed via agarose gel electrophoresis, and the remaining volume of the samples (20 μl each) were pooled. Pooled A domain and pooled 16S rRNA gene amplicons for each soil were purified using the QIAquick PCR purification kit (50) following the manufacturer’s instructions. Pooled KSI domain amplicons were gel purified using the QIAquick gel extraction kit (Qiagen) following the manufacturer’s instructions. Sequencing was performed by the NGS Competence Center Tübingen (NCCT) on a MiSeq system.

### Shotgun-seq analysis.

### (i) Shotgun metagenome analysis.

The shotgun Illumina and Nanopore reads were checked for sequence quality and adapter sequences using the FastQC tool. To assess the advantages of using both short and long reads for recovering metagenomic BGCs, we performed both individual technology-specific read assembly and hybrid assembly. Illumina reads were assembled using metaSPAdes (version 3.11.1) with default parameters (52). Hybrid assembly of Illumina and Nanopore reads were performed using metaSPAdes (53). Assembly comparisons were performed using the QUAST tool (54). Taxonomic annotation and abundance estimation analysis were performed both on reads and assembled contigs. Accelerated BLASTX annotations against the NCBI nonredundant protein database was done using Diamond (version 0.9.24) (55). The alignment-free fast taxonomic annotation tool Kraken2 with the maxikraken2 database (available from https://lomanlab.github.io/mockcommunity/mc_databases.html) was also used to annotate the taxonomy of reads and assembled metagenomes (56).

### (ii) Natural products biosynthesis domains and cluster annotation and diversity analysis.

Using the BiG-MEx tool, we performed BGC domain annotation and diversity analysis (57). Annotation of 150 domains involved in biosynthesis of natural products was done. The assembled contigs with lengths greater than 10 kb were run through a local installation of the antiSMASH pipeline (version 5) for identifying the BGCs (58). For more focused annotations of KS and C domains, the NaPDoS online server was used (3). BGCs were clustered using BiG-SCAPE with default parameters (59). GCFs containing MIBiG (version 2.0) BGCs were considered to be closer to known BGC products (60). The assembled contigs were also annotated using the DeepBGC tool to predict novel BGCs based on machine learning method (61).

### Amplicon-seq analysis.

### (i) Amplicon analysis (microbial abundance and diversity).

The QIIME 2 (version 2019.4) “Moving Pictures” tutorial steps were mostly followed for 16S amplicon analysis (62). DADA2 was used to process both sequencing reads, leading to longer amplicon sequence variants (ASVs) (63). The DADA2 pipeline performed quality filtering, denoising, and chimera detection (see Table S2 at https://doi.org/10.5281/zenodo.5195507). The ASVs were clustered into OTUs using the “vsearch” plugin available in QIIME2 based on 97% identity by the *de novo* clustering method. OTUs were classified using a naive Bayes classifier with the Silva database (version 132) (64). Subsequently, MAFFT-based multiple-sequence alignment of features was performed and used for phylogenetic tree construction via FastTree (65). q2-diversity plugin-based alpha diversity and beta diversity analyses were performed to compute Shannon, Faith PD, OTU, and evenness alpha diversity indices and Jaccard, Bray-Curtis, and UniFrac beta diversity distances.

### (ii) Amplicon analysis (BGC domain abundance).

Amplicons of the AMP-binding domain and the KS domain were analyzed using the QIIME2 pipeline steps described above for 16S amplicon analysis with modifications as described in the following text. Only read 1 sequences were used, as there was no overlap with read 2 and the relative quality of read 2 was poor. A hidden Markov model (HMM) search was performed using domain-specific HMM models available via the antiSMASH tool. Only the features matching the HMM models at default thresholds were further analyzed. ASVs were clustered at 97% identity using the q2-diversity plugin. KS domain sequence amplicons were further annotated using NaPDoS to identify putative pathway products. Domains matching with NaPDoS database domains with less than 85% identity were considered to be putative novel domains.

### (iii) Comparison of amplicon-seq and shotgun-seq identified BGC domains.

All of the shotgun-seq domains identified for each sample after the BiG-MEx analysis were concatenated. Using the “Dedupe” script from BBTools (version 37.62), domains were deduplicated at 85% identity. Amplicon-seq domains were mapped on the deduplicated domains from shotgun-seq using Burrows-Wheeler Aligner (BWA) and SAMtools to identify common and unique domains.

### Statistical analysis.

Spearman rank correlation was computed between alpha diversity indices of 16S, A domain, and KS domain. Similarly, correlation was also computed between alpha diversity indices and soil physicochemical parameters. R version 3.6.2 and RStudio were used to compute the statistical significance and correlation. The *ggplot2* package was used to develop the box plots (66). UpSet plots were developed using the UpSetR Shiny App webserver (67). The *qiime2* R package, Pavian (68), and the seaborn Python visualization library were used to plot the taxonomic profile and rarefaction curve.

### Data availability.

The sequencing data generated in this study were submitted to the NCBI Sequence Read Archive (SRA) and are accessible under BioProject identifiers PRJNA717813 and PRJNA717918. All relevant metadata, assembled contigs, annotated BGCs, and clustering results are available for download from https://doi.org/10.5281/zenodo.4644371.
